# The lipid flippase MoNeo1 mediates vesicle trafficking and pathogenicity in *Magnaporthe oryzae*

**DOI:** 10.1007/s44154-026-00305-5

**Published:** 2026-04-13

**Authors:** Yan Cai, Xiuwei Huang, Yufan Nie, Yueying Luan, Aarti Aarti, Qing Gong, Peng Sun, Yakubu Saddeeq Abubakar, Baohua Wang, Airong Wang, Guotian Li, Lili Lin, Wenhui Zheng

**Affiliations:** 1 State Key Laboratory of Agricultural and Forestry Biosecurity & Key Lab of Biopesticide and Chemical Biology, Ministry of Education, College of Plant Protection, Fujian Agriculture and Forestry University, Fuzhou, 350002 China; 2 State Key Laboratory for Biology of Plant Diseases and Insect Pests—Key Laboratory of Control of Biological Hazard Factors (Plant Origin) for Agri-Product Quality and Safety, Ministry of Agriculture, Institute of Plant Protection, Chinese Academy of Agricultural Sciences, Beijing, 100081 China; 3 Department of Biochemistry, Faculty of Life Sciences, Ahmadu Bello University, Zaria, 810281 Nigeria; 4 National Key Laboratory of Agricultural Microbiology, Hubei Hongshan Laboratory, Hubei Key Laboratory of Plant Pathology, The Center of Crop Nanotechnology, Huazhong Agricultural University, Wuhan, 430070 China

**Keywords:** Flippase, *Magnaporthe oryzae*, Pathogenicity, Vesicle transport

## Abstract

The rice blast fungus *Magnaporthe oryzae* poses a major threat to global rice production. In this study, we characterized MoNeo1, a P4-ATPase flippase, as a key regulator of fungal development and pathogenicity. Deletion of *MoNEO1* significantly impaired hyphal growth, conidiation, and appressorium function, and resulted in greatly reduced virulence. Lipidome profiling showed that MoNeo1 is essential for lipid homeostasis. The mutant exhibited significant accumulation of phosphatidylcholine, phosphatidylethanolamine, and phosphatidylserine, in addition to altered concentrations of phosphatidic acid and storage lipids. We further demonstrated that MoNeo1 physically interacts with the retromer subunit MoVps35, and that its subcellular localization at the interface between the trans-Golgi network and endosomes depends on retromer-mediated retrograde transport. Remarkably, MoNeo1 is required for the stability and proper transport of the SNARE protein MoSnc1, an important mediator of effector secretion. Our findings demonstrated MoNeo1 as a central hub that integrates lipid dynamics with vesicle transport to support fungal pathogenesis.

**Supplementary Information:**

The online version contains supplementary material available at 10.1007/s44154-026-00305-5.

## Introduction

The rice blast disease, caused by the fungus *Magnaporthe oryzae*, poses a significant threat to global food security. It leads to annual losses of around 6% of the global rice harvest, with epidemics often causing yield losses of up to 30% (Nalley et al. 2016; Savary et al. 2019). During infection, *M. oryzae* uses precise endosomal transport to facilitate its invasion and colonization of host plants (Chen et al. 2023, 2024; Oliveira‐Garcia et al. 2024; Zhang et al. 2024, 2025). Endosomal transport is a cornerstone of cellular function. It orchestrates the intricate ballet of molecular and organelle movement that maintains the integrity and functionality of the cell (Maxfield and McGraw 2004). Central to this system are the membrane-bound endosomes, which play a crucial role in regulating nutrient uptake and protein homeostasis (Cullen and Steinberg 2018). The process is initiated by endocytosis, whereby internalized molecules are channeled to early endosomes, which mature into late endosomes where their cargoes are sorted for subsequent degradation and/or recycling (Scott et al. 2014). The trans-Golgi network (TGN) is a master regulator that directs proteins to their final destinations and ensures the smooth operation of the cell machinery (Gu et al. 2001). The selective packaging and sorting signals of the TGN are crucial for precise protein delivery, which is essential for efficient cell function (Bard and Malhotra 2006; Baldridge and Graham 2012). In addition, endosomal transport regulates signal transduction and controls the fate of signaling molecules in a responsive cellular environment (Sorkin and von Zastrow 2009). However, the specific mechanisms by which endosomal transport regulates the pathogenicity of *M. oryzae* remain to be further elucidated.

Vesicle transport is a fundamental process in cells that ensures the precise delivery of cargo proteins and lipids to their correct destinations (Bonifacino and Glick 2004). Phospholipid flippases, particularly P4-ATPases, play a crucial role in this process by translocating phospholipids across membranes to generate lipid asymmetry (Andersen et al. 2016). This asymmetry is essential for various cellular functions, including the formation and budding of transport vesicles from the trans-Golgi network and endosomal membranes (Muthusamy et al. 2009). In yeast, Drs2-Cdc50 complex has been shown to play a key role in membrane trafficking and maintenance of lipid asymmetry (Timcenko et al. 2019). The structure and activation mechanism of this complex have been elucidated through cryo-EM studies, revealing its regulation by phosphatidylinositol 4-phosphate (PI4P) and other factors. Similarly, in humans, flippases like ATP8A1 are implicated in various cellular processes, and their dysregulation is linked to diseases such as cancer and neurological disorders (Van der Mark et al. 2013). Structural studies of human flippases have provided a better understanding of their function and potential therapeutic applications (Hiraizumi et al. 2019). In plant, previous studies have shown that the phosphatidylserine (PS) distribution established by Arabidopsis ALA3 is crucial for Rab GTPase-mediated vesicle trafficking and pollen tube growth (Poulsen et al. 2008). In the phytopathogen *Fusarium graminearum*, flippases like FgDnfA, FgDnfB, and FgDnfD play distinct roles in regulating vesicle transport and membrane lipid asymmetry, which are crucial for fungal development, pathogenicity and secondary metabolism (Yun et al. 2020). Compared to human and plant systems, the mechanism by which flippases integrate lipid dynamics with vesicle transport to support phytopathogen pathogenesis remains mysterious.

As a member of the flippase family, Neo1 plays a critical role in regulating membrane lipid asymmetry and vesicle transport. Neo1 was first identified as a neomycin resistance gene as it prevents the toxic effects of aminoglycoside antibiotics when it is overexpressed (Prezant et al. 1996). In *Saccharomyces cerevisiae*, Neo1 localizes to the Golgi and endosomal systems where it flips PS and phosphatidylethanolamine (PE) to maintain membrane integrity and facilitate vesicle trafficking (Takar et al. 2016). Mutations in *NEO1* disrupt membrane asymmetry and increase sensitivity to toxins (Wu et al. 2016). ATP9A and ATP9B, the mammalian homologs of Neo1, are localized to the Golgi and endosomal membranes (Yagi et al. 2025). ATP9A is required for the efficiency of endosomes-to-plasma membrane recycling pathway, contributing to membrane trafficking and vesicle formation (Tanaka et al. 2016). Additionally, ATP9B has been found to localize to the trans-Golgi network (TGN) in a CDC50 independent manner, indicating its role in vesicle transport and protein secretion (Takatsu et al. 2011). TAT-5, a *Caenorhabditis elegans* homolog of Neo1, is found in the plasma membrane. Inactivation of TAT-5 leads to specific exposure of PE in the outer leaflet of the plasma membrane (Wehman et al. 2011; Tanaka et al. 2016). These findings indicate that PE may be the primary substrate for Neo1/TAT-5/ATP9 subgroup of P4-ATPases. These studies underscore the conserved roles of Neo1 in membrane organization and cellular homeostasis across eukaryotes. However, the specific roles and functional mechanisms of Neo1 in pathogenic fungi remain in the dark.

This study explored the biological functions of MoNeo1 and the mechanism of its cellular trafficking function in *M. oryzae*. Results showed that *MoNEO1* deletion impaired mycelial growth, conidiation, and pathogenicity, highlighting its key role in fungal development and pathogenicity. Subcellular localization studies using the trans-Golgi marker MoKex2-mCherry and endosome dye FM4-64 revealed that MoNeo1-GFP co-localized with these markers, indicating that MoNeo1 localizes to the Golgi and endosome. Lipidomics analysis showed that deletion of *MoNEO1* impaired PS and PA levels. It was also found that MoNeo1 positively interacts with the retromer core subunit MoVps35, and this is crucial for the proper localization of MoNeo1. Furthermore, MoNeo1 mediates the transport of the SNARE protein MoSnc1 to the plasma membrane. These findings provide new insights into the intricacy of endosomal trafficking and sorting in plant-pathogenic fungi and offer a theoretical basis for eco-friendly disease management strategies.

## Results

### Identification and characterization of MoNeo1 in *M. oryzae*

**Fig. 1:**
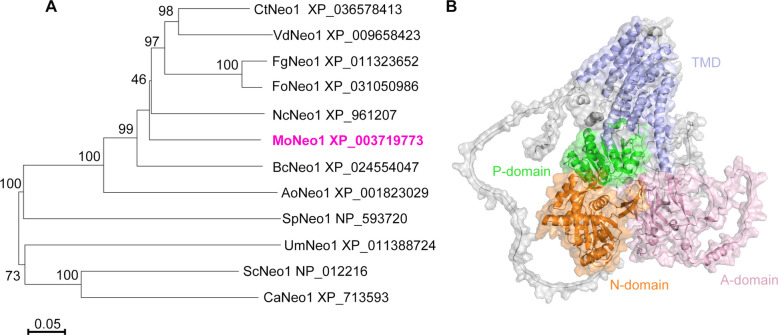
Phylogenetic analysis of the putative paralogue of Neo1 and 3D structure visualization of MoNeo1. **A** The cladogram illustrates the phylogenetic relationships of Neo1 across selected species. The maximum likelihood neighbor-joining phylogenetic tree was constructed using MEGA-X software. CtNeo1 (*Colletotrichum truncatum*), VdNeo1 (*Verticillium dahliae*), FgNeo1 (*F. graminearum*), FoNeo1 (*Fusarium oxysporum*), NcNeo1 (*Neurospora crassa*), MoNeo1 (*M. oryzae*), BcNeo1 (*B. cinerea*), AoNeo1 (*A. oryzae*), SpNeo1 (*Schizosaccharomyces pombe*), UmNeo1 (*Ustilago maydis*), ScNeo1 (*S. cerevisiae*), CaNeo1 (*Candida albicans*). **B** The 3D structure of MoNeo1, predicted by AlphaFold2, is displayed with a reported “plddts” value of 74.71

To identify the MoNeo1 protein in *M. oryzae* for subsequent functional characterization, the amino acid sequence of *S. cerevisiae* Neo1 (ScNeo1, NP_012216) was retrieved from the NCBI database and used for a BLAST search in the Kyoto Encyclopedia of Genes and Genomes (https://www.kegg.jp/kegg/). The results of the BLAST search identified the ScNeo1 ortholog MoNeo1 in *M. oryzae*, which is encoded by the gene MGG_04066. A detailed sequence analysis showed that the putative MoNeo1 is 55.28% identical to the ScNeo1 protein. The MoNeo1 sequence query covered 80% of the total length of ScNeo1 (Fig. S1). Corresponding results of phylogenetic analysis showed that Neo1 is phylogenetically conserved in filamentous fungi, and tightly clustered with BcNeo1 (XP_024554047) from *Botrytis cinerea* and AoNeo1 (XP_ 001823029) from *Aspergillus oryzae* (Fig. 1A). To further characterize the protein information of MoNeo1, its three-dimensional conformation was predicted using AlphaFold2. Domain annotations were made via the InterPro database (https://www.ebi.ac.uk), and structural insights were refined using spatial information from the homologous protein ScNeo1 (Jain et al. 2025). This analysis confirmed that MoNeo1 contains the classical P4-ATPase architecture, including the transmembrane domain (TMD), the actuator domain (A-domain), the nucleotide-binding domain (N-domain), and the phosphorylation domain (P-domain) (Fig. 1B). These comprehensive bioinformatic analyses reliably identify MoNeo1 as a conserved P4-ATPase in *M. oryzae* and provide a structural basis for further functional studies.

### MoNeo1 is localized to the TGN and endosomes in *M. oryzae*

**Fig. 2:**
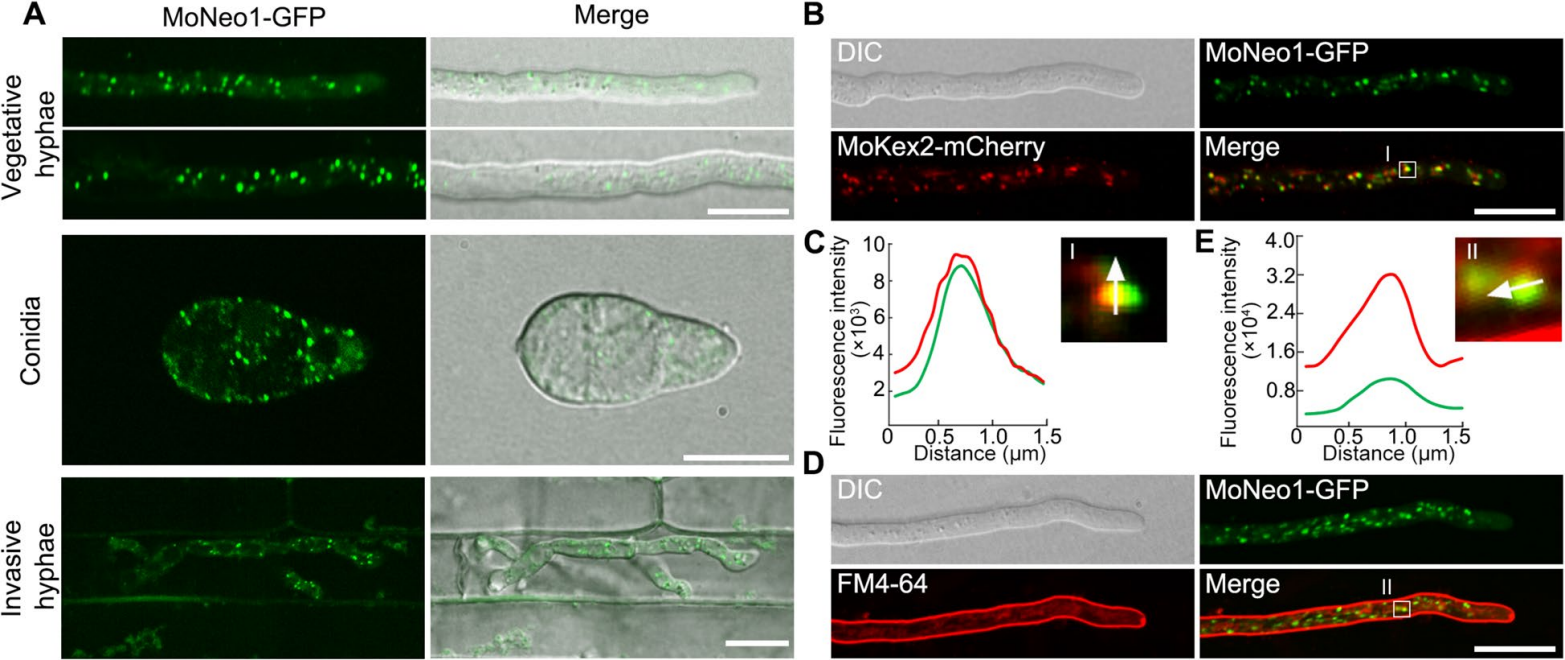
Subcellular localization of MoNeo1-GFP in *M. oryzae*. **A** Representative confocal micrograph of MoNeo1-GFP in the different developmental stages of *M. oryzae*. **B-C** MoNeo1-GFP exhibits partial co-localization with the TGN network marker MoKex2-mCherry in the vegetative hyphae of *M. oryzae*. **D-E** MoNeo1-GFP exhibits partial co-localization with the FM4-64 dye in the vegetative hyphae of *M. oryzae*. Scale bars = 10 μm. Each line scan graph was generated at the position indicated by the arrow in the zoomed regions to show the relative fluorescence intensity of the co-localization region

To elucidate the spatiotemporal dynamics of MoNeo1 during *M. oryzae* ontogeny, we performed live-cell fluorescence microscopy of a MoNeo1-GFP allele expressed under its native promoter. At the various developmental stages (vegetative growth, asexual development, invasive growth) of *M. oryzae*, MoNeo1-GFP consistently appeared as discrete and motile puncta (Fig. 2A and Movie S1). Considering that yeast Neo1 is known to cycle between the TGN and endosomal compartments (Dalton et al. 2017), we decided to interrogate the subcellular localization of MoNeo1. Co-expression of MoNeo1-GFP with the TGN marker MoKex2-mCherry showed partial co-localization in the fungal hyphae (Fig. 2B-C), indicating a TGN-associated localization. We then used FM4-64 dye to label the early endosomes and this showed transient co-localization of MoNeo1-GFP with the FM4-64-postive compartments, confirming an additional endosomal residence (Fig. 2D-E). Collectively, these data demonstrate that MoNeo1 is situated at the TGN-endosome interface and support its potential function in membrane trafficking during fungal development.

### MoNeo1 is involved in the vegetative growth and conidiation of *M. oryzae*

**Fig. 3:**
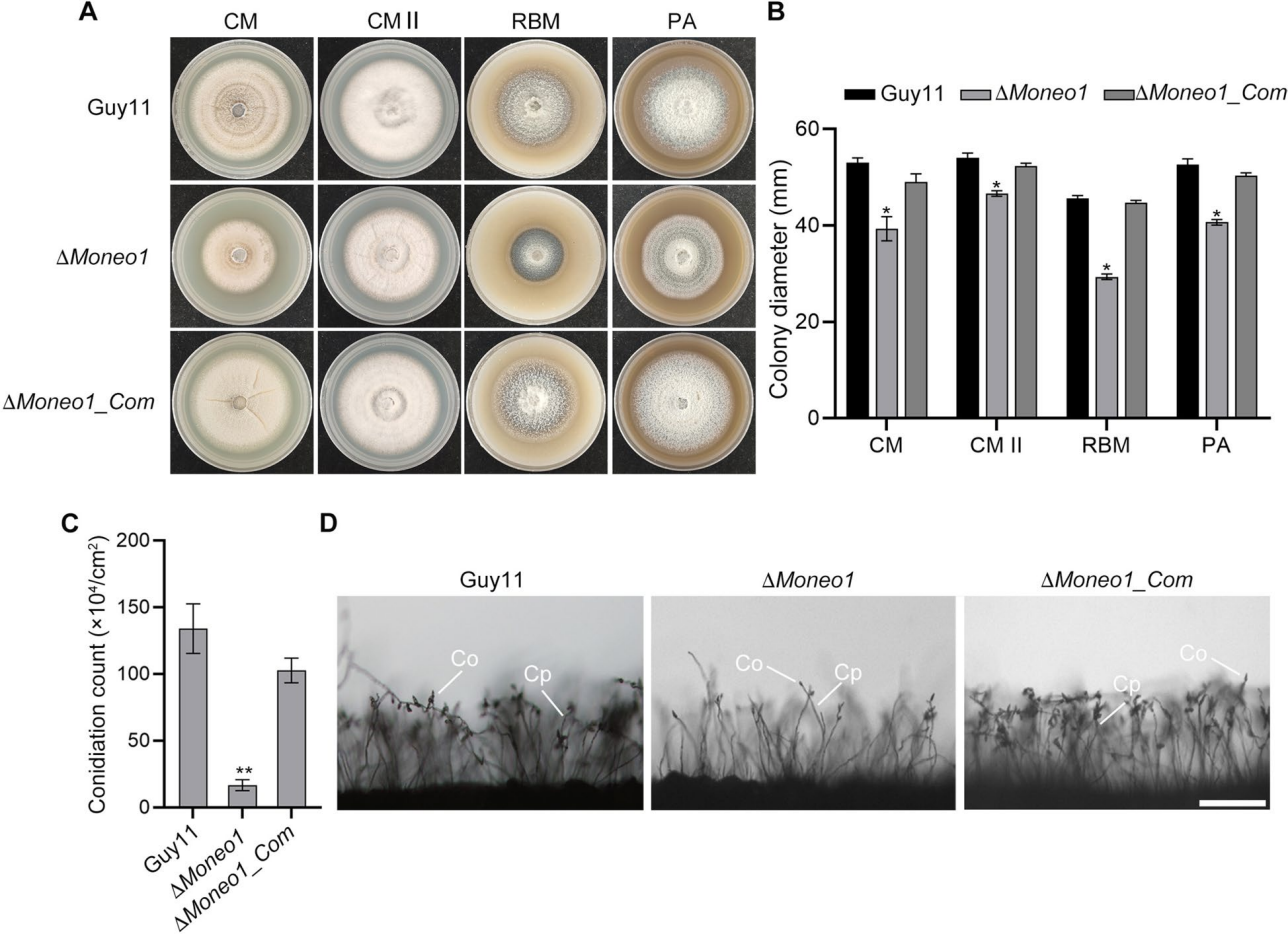
Role of MoNeo1 in vegetative growth and conidiation of *M. oryzae*. **A** Depicts the average vegetative growth of the Δ*Moneo1* strain, the complemented strain, and the wild-type strain cultured on CM, CMII, RBM, and PA after 10 days. **B** Represents the statistical analysis of vegetative growth records for the Δ*Moneo1* strain, the complemented strain, and the wild-type strain cultured on CM, CM II, RBM, and PA for 10 days. **C** Statistical analysis of conidiation in the Δ*Moneo1* strain, the complemented strain, and the wild-type strain. **D** Displays microscopic images comparing conidiophore formation in the Δ*Moneo1* strain, the complemented strain, and the wild-type strain under a 12-h light/dark cycle post condition after the vegetative growth phase. Co, conidia; Cp, conidiophore. Scale bar = 20 μm. Error bars represent the standard deviation between replicates, while “*” and “**” denote statistically significant at *P* ≤ 0.05 and *P* ≤ 0.01, respectively

To investigate the biological functions of MoNeo1 in *M. oryzae*, we used homologous recombination to generate the gene deletion mutant Δ*Moneo1* (Fig. S2). To assess the effect of *MoNEO1* gene deletion on the growth of *M. oryzae*, the wild-type Guy11, Δ*Moneo1* mutant, and Δ*Moneo1_Com* strains were cultured on complete media (CM), CM II, rice bran media (RBM), and plum agar media (PA) and incubated for 7 days at 28℃ under 12 h light/dark conditions. The growth assay showed that the Δ*Moneo1* mutant exhibited significantly stunted growth compared to the wild-type and complemented strains (Fig. 3A-B). Sporulation is critical for the persistence and spread of rice blast disease. To clarify the possible contribution of MoNeo1 to asexual reproduction, we monitored conidiation and conidiophore formation. We found that deletion of *MoNEO1* impaired conidiophore development and drastically reduced conidia production in Δ*Moneo1* strains (Fig. 3C-D). These results suggest that MoNeo1 has a regulatory effect on hyphal growth and asexual reproduction in *M. oryzae*.

### MoNeo1 contributes to the full virulence of *M. oryzae*

**Fig. 4:**
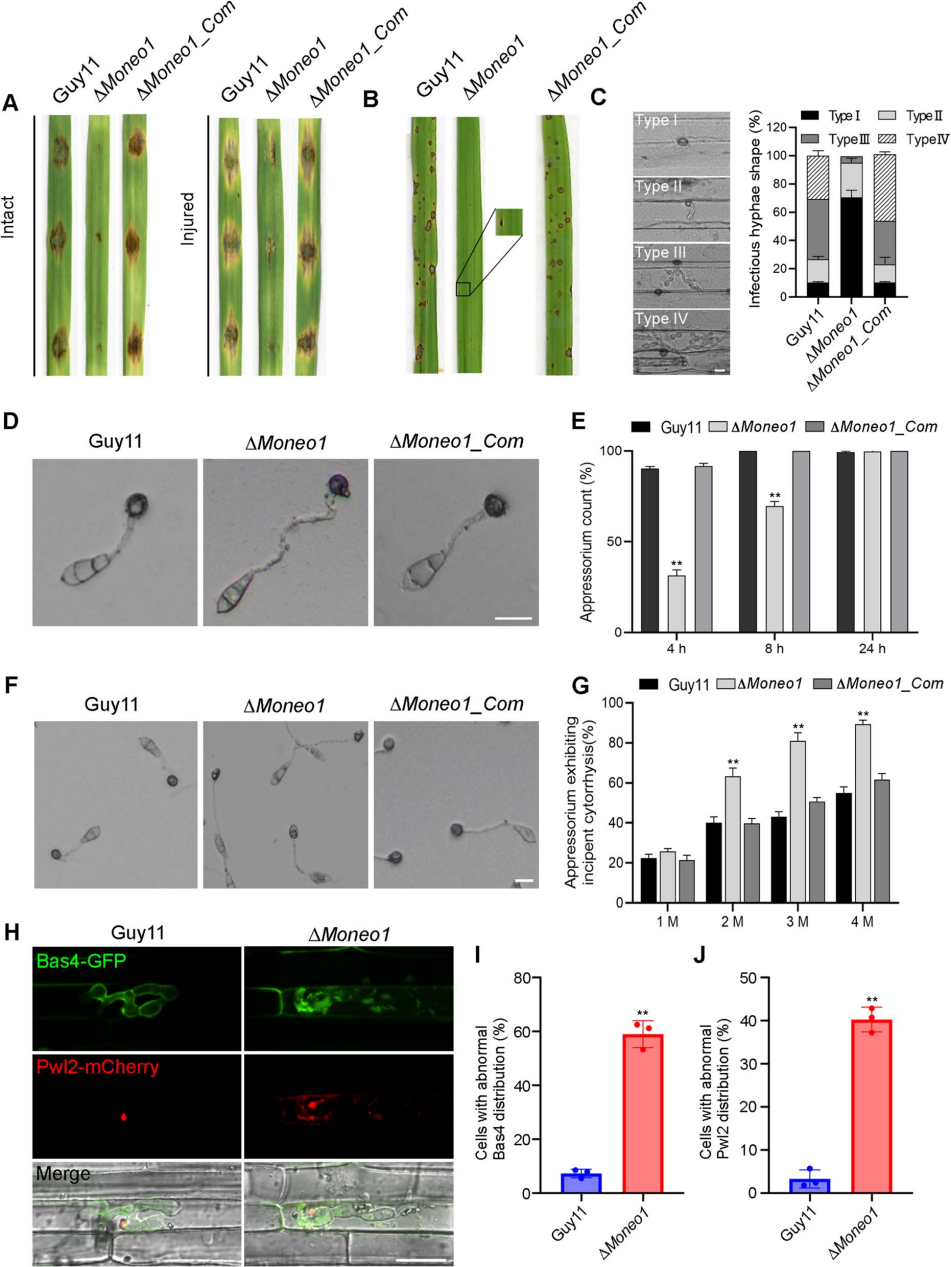
MoNeo1 impacts the pathogenicity of rice blast fungus. **A** Spore suspension inoculation on detached barley leaves. **B** Virulence assessment of the Δ*Moneo1* strain, the complemented strain and the wild-type strain on 3-week-old susceptible CO39 rice seedlings inoculated with spore suspensions. **C** Comparative quantitative analyses of the invasive development of the individual strains were classified into four types: type I (appressorium), type II (primary invasive hyphae), type III (invasive hyphae), and type IV (with extensive hyphal growth). Statistical evaluations were performed with almost consistent values obtained from three biological experiments with three technical replicates each time. (*n* = 300 infection sites scanned per replicate). **D** Micrograph portrays appressorium formation in the Δ*Moneo1* inoculated on hydrophobic slides 8 hpi compared with the wild-type and complemented strains. **E** Bar graph shows statistical computation of records obtained from microscopy examination of the appressorium formed by conidia from the wild-type, Δ*Moneo1* and complemented strains on hydrophobic coverslips at 4,8 and 24 hpi. **F** Cytorrhysis assays measuring appressorium turgor in the Δ*Moneo1* strain, the complemented strain and the wild-type strain on hydrophobic coverslips after an 8-h incubation in 2 M glycerol. **G** Statistical analysis of appressoria treated with glycerol solutions (1, 2, 3, and 4 M) and observed via a Nikon light microscope. **H** Fluorescence microscopy showing the effector proteins Bas4 (apoplastic effector) and Pwl2 (cytoplasmic effector) distribution in the wild-type strain and Δ*Moneo1*. **I-J** Bar charts illustrating the percentage of abnormal distribution of Bas4 and Pwl2 in the wild-type strain and Δ*Moneo1*. Scale bars = 20 μm. Error bars represent the standard deviation between replicates, “**” denotes statistical significance at *P* ≤ 0.01

To investigate the pathogenicity and virulence efficiency of the conidia produced by Δ*Moneo1*, we conducted inoculation experiments on both intact and injured barley leaves by drop-inoculation using spores suspension from the various strains, and on susceptible rice seedlings CO39 by spray-inoculation with spores of each strain. The results showed that deletion of *MoNEO1* significantly reduced the ability of conidia to induce blast symptoms on both intact and injured leaf tissue (Fig. 4A-B). In addition, the conidia were also injected into the leaf sheaths of rice. The results showed that the disruption of *MoNEO1* strongly compromised host both penetration and colonization efficiency (Fig. 4C). To investigate the reduced pathogenicity of the Δ*Moneo1* mutant, we examined appressorium formation on artificial hydrophobic surfaces. After 4- and 8-h of incubation, we found that the *MoNEO1* deletion mutant exhibited elongated germ tubes but no significant difference in appressorium morphogenesis compared to the wild-type and complemented strains (Fig. 4D). Appressoria formation in the Δ*Moneo1* mutant was significantly reduced by more than 50% of the wild-type and complemented strains. However, after extending incubation to 24 h, the appressorium formation of the Δ*Moneo1* mutant was up to the level of the wild-type and complemented strains (Fig. 4E). Given that appressorium turgor pressure is critical for host penetration, we measured the appressorial turgor using cytorrhysis assays. After treating 8-h-old appressoria with difference concentrations (1, 2, 3, and 4 M) of glycerol, we observed significantly higher appressorial collapse rates in the Δ*Moneo1* mutant compared to the wild-type and complemented strains (Fig. 4F-G). This indicates reduced turgor in the Δ*Moneo1* mutant appressoria. Cosistent with this, Nile red straining revealed impaired lipid retention at 6 h and 16 h post-induction (Fig. S3), suggesting defective glycerol generation. Collectively, these findings suggest that MoNeo1 plays a crucial role in appressorium turgor generation in *M. oryzae*.

Given that MoNeo1 localizes to the trans-Golgi network and is implicated in vesicle trafficking, we investigated whether effector secretion is compromised in the Δ*Moneo1* mutant. We examined the secretion of two effectors, Bas4 and Pwl2, fused to GFP and mCherry respectively, during host cell invasion. In the Δ*Moneo1* mutant, Pwl2-mCherry formed multiple cytosolic foci and Bas4 accumulated in the cytosol and vacuole, indicating that MoNeo1 is required for proper effector trafficking and secretion (Fig. 4H-J). These findings indicate that MoNeo1-mediated vesicle trafficking is essential for effector secretion and translocation into host cells, which likely contributes to the reduced virulence observed in the mutant.

### MoNeo1 contributes to lipid metabolism in *M. oryzae*

**Fig. 5:**
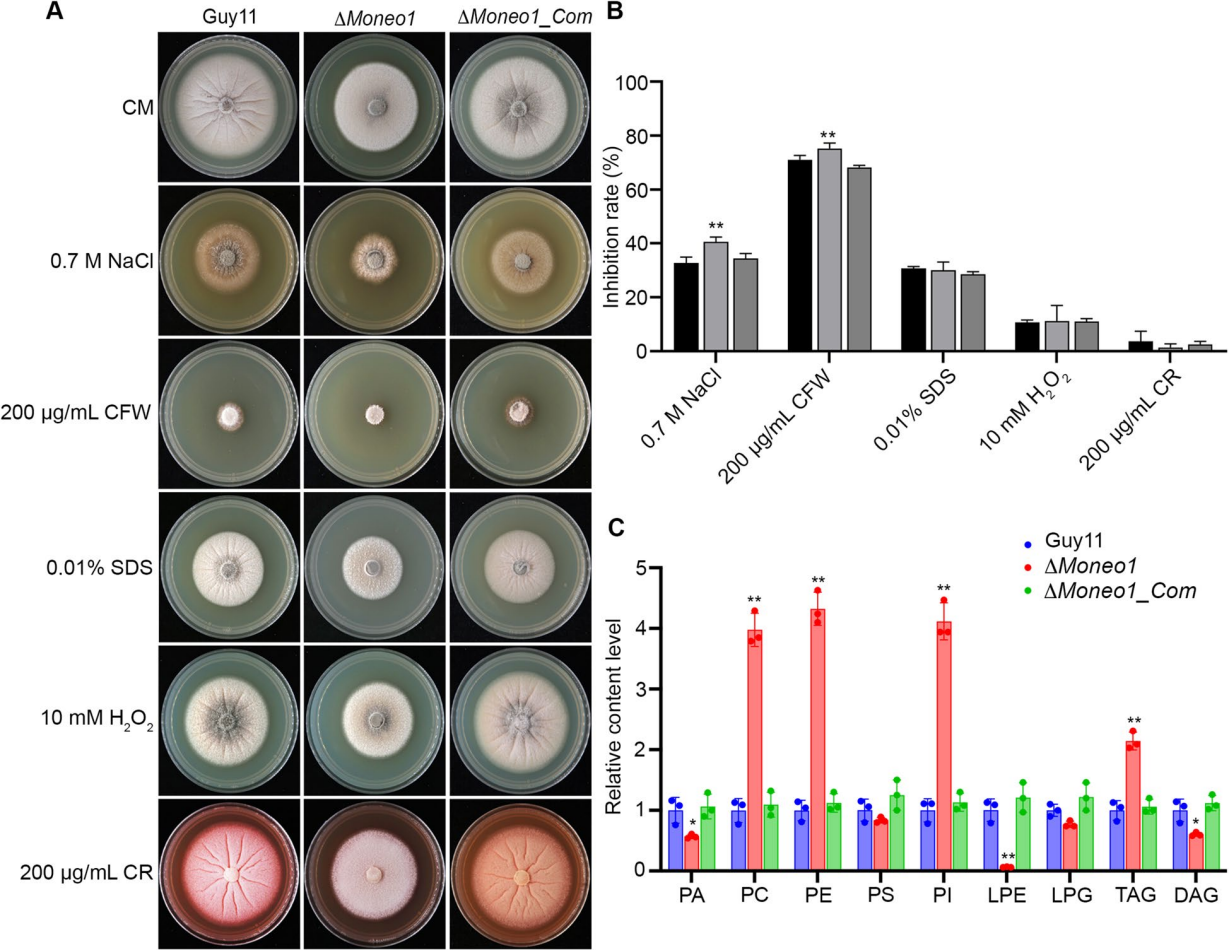
Lipid composition changes in Δ*Moneo1*. **A** Showed the physical inhibitory effect of selected cell membrane and cell wall stress inducing osmolytes on the vegetative growth of Δ*Moneo1*, the complemented and wild-type strains cultured on CM media supplemented with either 2 mM DTT, 0.7 M NaCl, 200 μg/mL Calcofluor White (CFW), 0.01% SDS, 10 mM H_2_O_2_ or 200 μg/mL Conge Red (CR) for 10 days. **B** Represent statistical evaluation of the response of *MoNEO1* deletion mutant, the wild-type strain to different cell membrane/wall stress inducing osmolytes. The inhibition data was generated from three independent biological experiments with five technical replicates each time. *t* test statistical analysis was carriedout with Microsoft Excel spreadsheet and error bars represent the standard deviation. Inhibition rate = (the diameter of untreated strain − the diameter of treated strain)/(the diameter of untreated strain) × 100%. **C** Relative lipid contents of PA (phosphatidic acid), PC (phosphatidylcholine), PE (phosphatidylethanolamine), PS (phosphatidylserine), PI (phosphatidylinositol), LPE (lysophosphatidylethanolamine), LPG (lysophosphatidylglycerol), TAG (triacylglycerol), and DAG (diacylglycerol) in Guy11, Δ*Moneo1*, and Δ*Moneo1_Com* strains. Error bars represent the standard deviation between replicates. Error bars represent the standard deviation between replicates, “*” denotes statistical significance at *P* ≤ 0.05, “**” denotes statistical significance at *P* ≤ 0.01

Previous studies have demonstrated that flippases are implicated in the maintenance of PS asymmetry across cell membranes (Hankins et al. 2015). As PS constitutes a vital component of the cell membrane and serves as a sensor for environmental fluctuations, we decided to investigate the effect of *MoNEO1* deletion on the fungal growth under cell membrane and cell wall stress conditions. We compared the phenotype of Δ*Moneo1* mutant with the complemented and wild-type strains under the stress conditions. Results showed that the Δ*Moneo1* mutant exhibited increased susceptibility to NaCl- and CFW-induced membrane stress compared to the wild-type and complemented strains (Fig. 5A and B). These findings suggest that MoNeo1 is important for maintaining membrane integrity and fluidity in *M. oryzae*.

Considering the involvement of MoNeo1 in membrane trafficking and its localization to TGN-endosome interface, we hypothesized that it might influence lipid homeostasis. To test this, we performed lipidomics analysis by high-performance liquid chromatography-mass spectrometry (HPLC–MS) to quantify the relative lipid content in Guy11, Δ*Moneo1,* and Δ*Moneo1_Com* strains. Consistent with the earlier findings, the Δ*Moneo1* mutant exhibited significant alterations in lipid composition compared to Guy11. Specifically, the levels of phosphatidylcholine (PC), PE, PS, and phosphatidylinositol (PI) were elevated in the mutant by 42%, 38%, 21%, and 19%, respectively, while PA and lysophosphatidylethanolamine (LPE) levels decreased by 28%, and 17%, respectively (Fig. 5C). Additionally, in the mutant, the levels of triacylglycerol (TAG) and diacylglycerol (DAG) increased by 23% and 14%, respectively (Fig. 5C). These changes were largely reversed in the complemented strain Δ*Moneo1_Com* (Fig. 5C), underscoring the crucial role of MoNeo1 in maintaining lipid homeostasis. The observed lipid profile shifts suggest that MoNeo1 influences membrane fluidity and integrity through modulating the levels of key phospholipids, leading to broader implication in cellular processes including membrane trafficking and pathogenicity.

### Retromer regulates the spatial distribution of MoNeo1 in *M. oryzae*

**Fig. 6:**
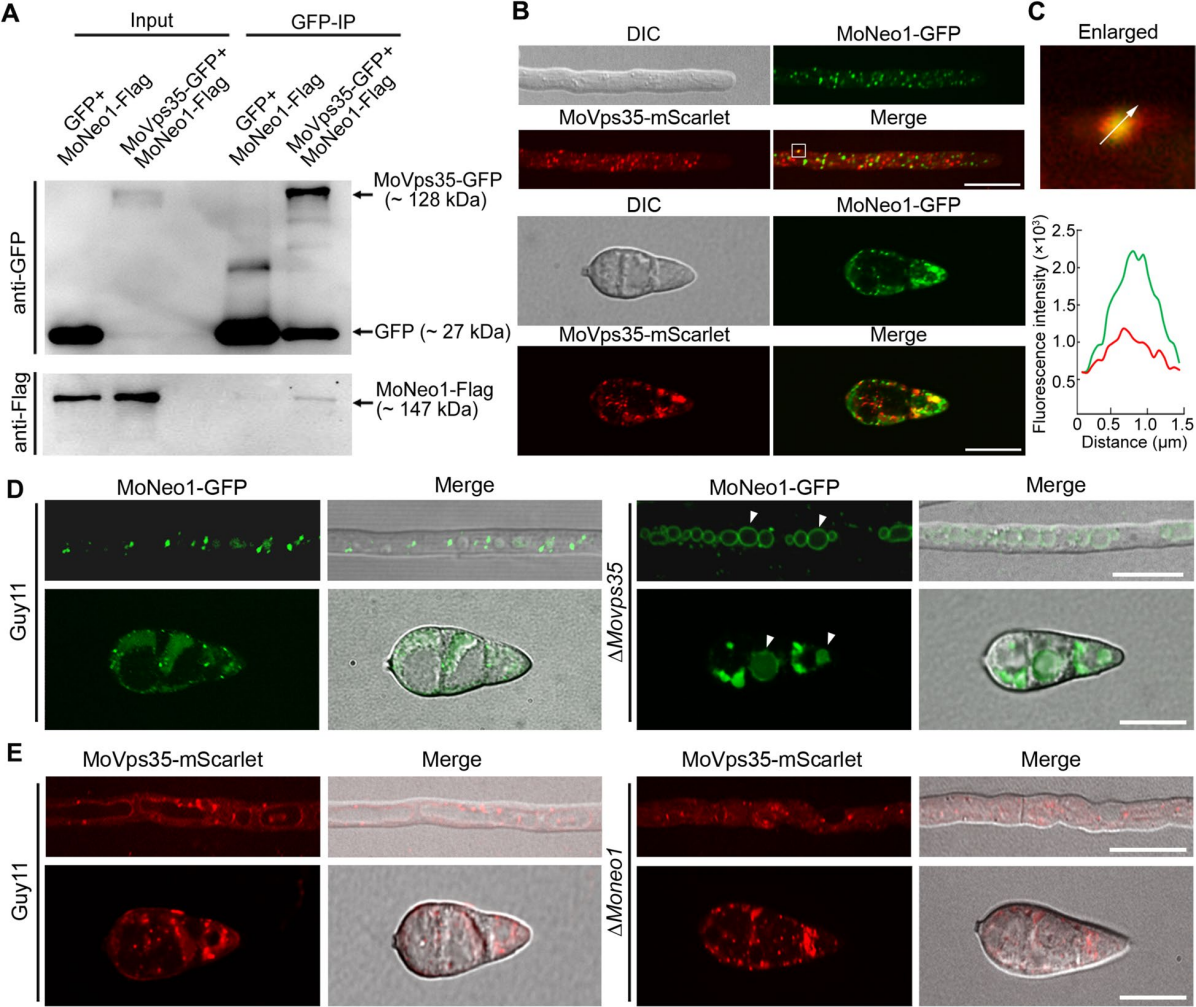
Retromer-dependent trafficking governs the subcellular localization of MoNeo1. **A** GFP-Trap pull-down demonstrating interaction between MoNeo1 and the retromer core subunit MoVps35. **B** Co-localization of MoNeo1-GFP with MoVps35-mScarlet in vegetative hyphae and conidia stages reveals overlapping puncta. **C** Fluorescence intensity line scan along the arrow in the enlarged inset quantifies the degree of co-localization shown in enlarged zoom. **D** Deletion of *MoVPS35* displaces MoNeo1-GFP from its native TGN/endosome puncta to the vacuolar membrane, whereas MoNeo1-GFP maintains its punctate distribution in the wild-type strain background. Arrows indicate vacuolar membranes. **E** Deletion of *MoNEO1* does not perturb the punctate localization of MoVps35-mScarlet relative to the wild-type control. Scale bars = 10 µm

Having established that MoNeo1 resides at the TGN-endosome interface, we wondered how its steady-state distribution is maintained. Retromer is a conserved pentameric complex that selectively retrieves membrane cargoes from endosomes to the TGN, thereby preventing their lysosomal/vacuolar degradation (Tu and Seaman 2021). Neo1 has been identified as a retromer cargo in yeast (Dalton et al. 2017). However, whether MoNeo1, which exhibits partial TGN localization (Fig. 2), has similar functions in *M. oryzae* remains unclear. To test this hypothesis, we co-expressed MoNeo1-flag with MoVps35-GFP, the core retromer cargo-recognition subunit. Co-immunoprecipitation assay recovered MoNeo1-flag with MoVps35-GFP but not free GFP control, confirming a positive interaction in vivo (Fig. 6A). Moreover, live-cell imaging revealed that MoNeo1-GFP partially co-localized with MoVps35-mScarlet on motile puncta that traverse the hyphal cytoplasm (Fig. 6B-C). To determine whether the retromer mediates the subcellular localization of MoNeo1, we monitored the dynamics of MoNeo1-GFP signal in *MoVPS35* deletion mutant background. Interestingly, the TGN-endosome punctate localization of MoNeo1-GFP was not detectable in the absence of MoVps35; instead, MoNeo1-GFP accumulated on vacuole membrane both in vegetative hyphae and conidia (Fig. 6D). In contrast, the normal distribution of MoVps35-GFP remained unchanged in the absence of MoNeo1 (Fig. 6E), implying that retromer upstream of MoNeo1 in the retrograde trafficking pathway. Collectively, these data suggest that MoNeo1 remains a retromer cargo in *M. oryzae*.

### MoSnc1 as a cargo in the flippase/retromer-mediated trafficking pathway

**Fig. 7:**
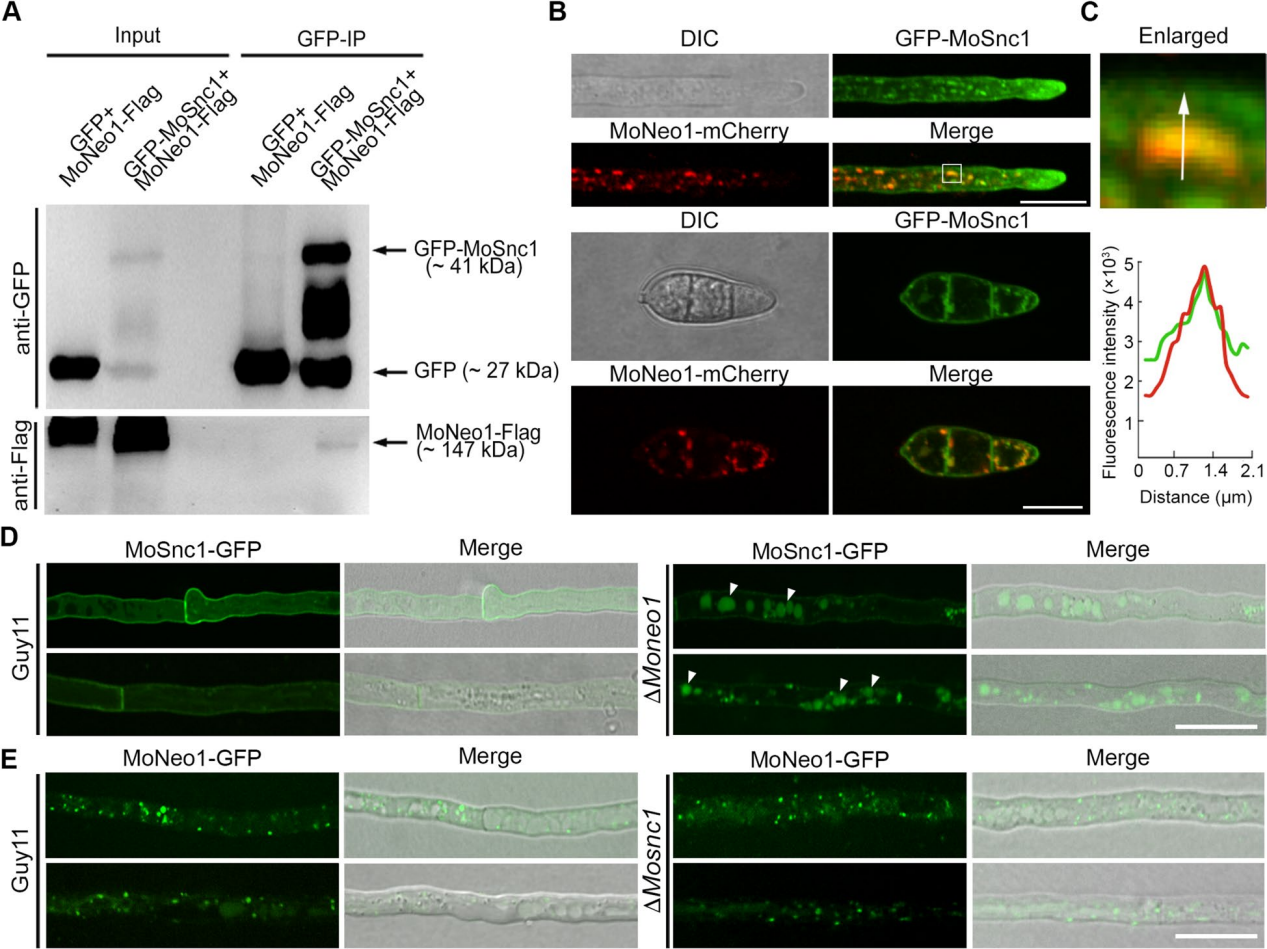
MoNeo1 interacts with the retromer cargo MoSnc1. **A** GFP-Trap pull-down experiment demonstrating the physical interaction between MoNeo1 and the R-SNARE protein MoSnc1. **B** Co-localization of MoNeo1-mCherry with GFP-MoSnc1 in vegetative hyphae and conidia, revealing overlapping punctate structures. **C** Fluorescence intensity line scan along the arrow in the enlarged inset quantifies the degree of overlap shown in B. **D** Deletion of *MoNEO1* causes GFP-MoSnc1 to mislocalize to vacuole lumen (arrows). The vacuole lumen can be identified by its concave and circular pattern in the merged channel. **E** Deletion of MoSNC1 does not perturb the punctate localization of MoNeo1-GFP relative to the wild-type control. Scale bars = 10 µm

Building on our findings that MoNeo1 localizes to the TGN-endosome interface and interacts with the retromer subunit MoVps35, we explored the functional relationship between MoNeo1 and the retromer complex. Given that the retromer mediates retrograde trafficking of cargo proteins from endosomes to the TGN and flippases like MoNeo1 are essential for maintaining membrane fluidity and integrity, we hypothesized that MoNeo1 might regulate the trafficking of specific retromer cargoes. To identify potential cargoes involved in the flippase/retromer-mediated trafficking pathway in *M. oryzae*, we examined our LC–MS/MS data from MoNeo1-Flag pull-down experiments which identified a total of 521 proteins (Table S2). After refining the list by selecting those proteins that appeared in MoNeo1-Flag but not in the controls, and considering only those with sequence coverage ≥ 15%, we identified the SNARE protein MoSnc1 (MGG_12614), a known retromer cargo localized to endosome/plasm membrane and involved in effector secretion in *M. oryzae* (Chen et al. 2023) (Fig. S4). Co-immunoprecipitation (Co-IP) assays confirmed interaction between MoSnc1 and MoNeo1 (Fig. 7A). Furthermore, live-cell imaging showed partial co-localization of GFP-MoSnc1 with MoNeo1-mCherry, suggesting that they are potentially co-transported (Fig. 7B). To determine whether flippase/retromer trafficking pathway contributes MoSnc1 localization, we examined the subcellular localization of GFP-MoSnc1 in Δ*Moneo1* mutant as well as that of MoNeo1-GFP in Δ*Mosnc1* mutant. In Δ*Moneo1*, the number and sizes of GFP-MoSnc1 puncta were obviously reduced, with some being mis-sorted to vacuoles in older hyphae and conidia (Fig. 7C). Conversely, MoNeo1-GFP localization remained unaffected in Δ*Mosnc1* mutants (Fig. 7D). Collectively, these results demonstrate that MoNeo1 is crucial for proper localization of MoSnc1, highlighting its role in regulating vesicle trafficking and cargo distribution in *M. oryzae*.

## Discussion

**Fig. 8:**
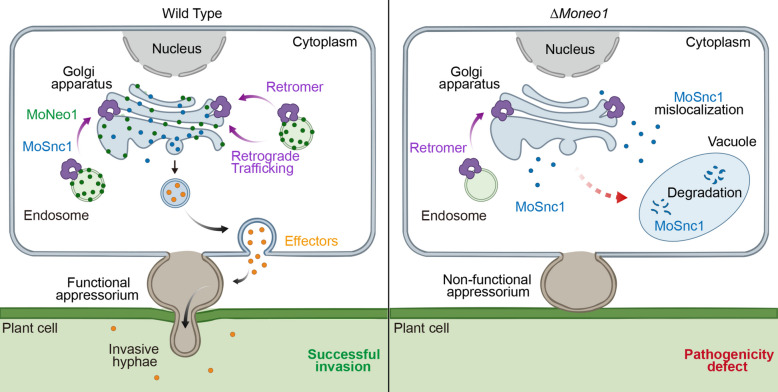
A working model of MoNeo1 in pathogenicity of *M. oryzae.* In the wild-type strain (left), the retromer complex retrieves MoNeo1 from endosomes to the TGN, preventing its degradation and maintaining its steady-state localization. At the TGN/endosome interface, the lipid environment established by MoNeo1 is essential for the efficient trafficking and stability of the SNARE protein MoSnc1, enabling its proper function in vesicle fusion and effector secretion, which supports functional appressorium development and successful host invasion. In contrast, in the Δ*Moneo1* mutant (right), the absence of MoNeo1 disrupts this pathway, MoSnc1 fails to localize correctly, undergoing mislocalization and vacuolar degradation. This disruption in vesicular trafficking ultimately leads to non-functional appressoria and defect in pathogenicity

Vesicle transport is a tightly regulated cellular process requiring precise coordination of cargo recruitment, coatomer assembly, and lipid dynamics, and is facilitated by phospholipid flippases (Muthusamy et al. 2009). As members of the P4-ATPase family, flippases translocate phospholipids from luminal to cytosolic leaflet of membranes (Daleke 2007). This activity is essential for maintaining lipid asymmetry and it supports membrane trafficking through the Golgi apparatus and endosomal system (Sebastian et al. 2012). As a flippase, Neo1 plays a key role in establishing aminophospholipid asymmetry in the plasma membrane, thereby facilitating proper membrane trafficking and vesicle formation within the endomembrane system (Wu et al. 2016; Tanaka et al. 2016). In this study, we sought to elucidate the role of the flippase MoNeo1 in fungal pathogenesis and to delineate its regulatory pathway. We demonstrated that MoNeo1 is involved in vegetative growth, conidiation, appressorium formation and pathogenicity of *M. oryzae*. Furthermore, MoNeo1 acts as a downstream cargo of the retromer and contributes to the proper trafficking of MoSnc1 (Fig. 8).

The phenotypic spectrum resulting from Neo1 depletion varies significantly across eukaryotes. In *S. cerevisiae*, *NEO1* deletion is lethal, underscoring its fundamental role in maintaining basic cellular functions (Hua et al. 2002). This is contrary to observations in filamentous fungi, including *F. graminearum* and *A. nidulans*, where loss of *DNFD*, the *NEO1* ortholog, yields viable mutant though with impaired asexual differentiation (Schultzhaus et al. 2019; Yun et al. 2020). Also, asexual differentiation was severely disrupted. Our findings in *M. oryzae* reveal a distinct and more severe phenotypic features. Although MoNeo1 is dispensable for viability, its deletion resulted in significantly suppressed vegetative growth. Furthermore, asexual sporulation was drastically compromised, with conidia production reduced by approximately 87.3% (Fig. 3). The phenotypic disparity between yeast and filamentous fungi suggests the existence of compensatory mechanisms or genetic redundancy in more complex organisms. Notably, the essentiality of Neo1 appears to be more heightened in higher eukaryotes. In humans, mutation in the Neo1 ortholog ATP9A causes an autosomal recessive hypotonia, intellectual disability (ID), and attention deficit hyperactivity disorder (ADHD), demonstrating its critical role in development (Meng et al. 2023). This cross-kingdom comparison positions *M. oryzae* as an intermediate model where MoNeo1 is dispensable for survival but essential for development and asexual production. This highlights an indispensable and specific role for Neo1 in mediating genetic pathways essential for asexual differentiation.

P4-ATPase flippases have been established as critical virulence factors in a broad spectrum of fungal pathogens, governing essential pathogenic processes such as polarized growth, vesicle-mediated trafficking, and the development of specialized infection structures (Balhadère and Talbot 2001; Hu and Kronstad 2010; Rizzo et al. 2014). In the wheat scab pathogen *F. graminearum*, the targeted disruption of the *NEO1* ortholog *FgDNFD* has been demonstrated to significantly impair its pathogenicity on wheat heads (Yun et al. 2020). Similarly, in the human pathogen *C. neoformans,* the Drs2 homolog Apt1 is indispensable for survival and proliferation within mammalian hosts (Rizzo et al. 2014). This conserved flippase-dependent pathogenesis also holds true in *M. oryzae* in which the Drs2 homolog MoPde1 was shown to be essential for appressorium-mediated host penetration (Balhadère and Talbot 2001). Our study identified MoNeo1 as another pivotal flippase crucial for the pathogenesis of *M. oryzae*. The complete loss of invasive hyphae in the Δ*Moneo1* mutant provides compelling phenotypic evidence of its essential role. Mechanistically, we attribute this virulence defect to failure in appressorium function; despite eventual formation, these structures are bioenergetically incompetent, unable to generate the immense turgor pressure required for mechanical breaching of the host cuticle (Fig. 4).

During membrane transport from the Golgi to the plasma membrane, flippases selectively translocate PS and PE from the luminal or extracellular leaflet to the cytosolic leaflet to maintain membrane asymmetry (Norris et al. 2024). Neo1, a Golgi-localized PS/PE flippase, is essential for establishing PS/PE asymmetry in the plasma membrane (Takar et al. 2016; Bai et al. 2021). The lipidomic profile of the Δ*Moneo1* mutant underscores a fundamental role for MoNeo1 in governing phospholipid homeostasis in *M. oryzae*. The observed accumulation of PS, PE, PC, and PI in the Δ*Moneo1* mutant suggests a conserved function where impaired lipid flipping disrupts the metabolic and spatial distribution of key phospholipids. This is likely compounded by defective TGN-/endosomal-associated vesicle trafficking, a process known to be regulated by flippase activity in other systems (Sebastian et al. 2012). The concomitant decrease in PA, DAG, and TAG points to a broader dysregulation of lipid metabolic pathways. A similar shift toward neutral lipid storage has been observed in other lipid trafficking mutants and is often associated with disruption in the utilization of phospholipid precursors, reflecting a conserved metabolic response to membrane homeostasis stress (Kohlwein 2010). This metabolic rerouting may represent a compensatory cellular response to membrane bilayer stress or an imbalance in lipid signaling.

Our results define a critical functional axis coordinating retrograde protein sorting with lipid translocation in *M. oryzae*, revealing how the retromer complex and the flippase MoNeo1 jointly govern vesicle transport to support pathogenesis. While the retromer-dependent retrieval of MoNeo1 from endosomes to the TGN is consistent with findings in model yeasts (Dalton et al. 2017), the functional impact of MoNeo1 on a virulence-associated SNARE protein underscores its specialized role in the plant pathogen. MoSnc1 is a key mediator of effector secretion (Chen et al. 2023). The observed mis-localization and vacuolar degradation of MoSnc1 in Δ*Moneo1* mutant demonstrates that MoNeo1 activity is indispensable for the correct membrane partitioning of this key secretion-related SNARE. This dependency suggests that MoNeo1-generated membrane asymmetry may facilitate the formation of specific lipid domains that are necessary for stability, fusion competence or recycling of MoSnc1-containing transport intermediates (Pomorski and Menon 2006). Such a role is consistent with emerging evidence that phospholipid asymmetry directly modulates SNARE complex assembly and function by altering the physicochemical properties of membranes and exposing phosphatidylserine residues required for efficient membrane fusion (Takamori et al. 2006; Murray and Tamm 2011). The specific mistargeting of a retromer cargo upon loss of MoNeo1 further implies that certain retrograde trafficking steps rely on flippase-mediated lipid remodeling, potentially for the formation or scission of retromer-coated vesicles (Kvainickas et al. 2017; Simonetti et al. 2019). Establishing whether the flippase activity of MoNeo1 is mechanistically required for MoSnc1 trafficking rather than serving a purely structural role will clarify how lipid-dependent sorting is biochemically achieved during infection-related development in fungal pathogens.

## Conclusions

In this study, we identified the P4-ATPase flippase MoNeo1 as a key regulator of growth, development, and pathogenicity in *M. oryzae*. We demonstrated that MoNeo1 maintains phospholipid asymmetry and lipid homeostasis and established that its TGN/endosome localization is mediated by the retromer complex. Importantly, MoNeo1 is required for the proper trafficking of the SNARE protein MoSnc1, functionally coupling lipid translocation to vesicular transport and effector secretion during fungal infection. Our findings uncover a crucial mechanism by which MoNeo1 integrates membrane lipid dynamics with retrograde trafficking to support virulence, highlighting its role as a central coordinator of infection-related development in this major plant pathogen.

## Methods

### Strains and culture conditions

The *M. oryzae* wild-type strain Guy11 was used to generate *MoNEO1* deletion mutants. Genetically manipulated strains were cultured on CM plates (6 g yeast extract, 6 g casein hydrolysate, 10 g sucrose, and 20 g agar per liter) at 28 °C. Liquid CM was used for mycelia growth for DNA extraction and protoplast preparation. For conidiation assay, strains were cultured on RBM plates (40 g rice bran and 20 g agar per liter at pH 6), or CM II plates (10 g D-glucose, 2 g peptone, 1 g yeast extract, 1 g casamino acids, 50 mL 20 × nitrate salts, 1 mL trace elements, 1 mL vitamin solution, and 15 g agar per liter at pH 6.5).

### Generation of gene deletion mutants and complementation

To generate Δ*Moneo1* mutants, targeted gene deletion by homologous recombination was performed. Approximately 1 kb DNA sequences upstream and downstream of the targeted open reading frames (ORFs) were amplified and cloned into a pCX62 vector harboring hygromycin resistance gene for selection. The resulting plasmids were used to generate AH and HB fusion fragments, which were then amplified and introduced into the protoplast of the wild-type strain. Initial screening of transformants was done using PCR using OF/OR and UF/H853 primer pairs, followed by Southern blot analysis for verification.

For constructing the GFP-fusion vector, the coding sequence of the targeted gene was amplified from Guy11 genomic DNA using Gene-GF and Gene-GR primers and cloned into the pKNTG vector using One Step Cloning Kit (Vazyme Biotech Co., Ltd), with sequence analysis confirming the construct. Other fusion vectors were constructed similarly. Correct insertion and integrity of the inserted fragments were confirmed by sequencing, and the vectors were transformed into the mutants’ protoplasts by PEG-mediated transformation. All the primers used are listed in Table S1.

### Assays for vegetative growth and conidiation

Both the wild-type and the genetically modified strains, including mutants and complemented lines, were cultivated on four distinct media: CM, PA (40 mL prune juice, 5 g lactose, 5 g sucrose, 1 g yeast extract, and 20 g agar per liter, pH 6.5), CM II, and RBM, at 28 °C with 12 h light–dark cycles for vegetative growth assessment. Colony diameters were measured after 10 days post incubation.

For the conidiation assay, the strains were cultured in continuous darkness on RBM for 7 days. The hyphae were carefully scraped with sterilized glass slides to detach the conidia. They were then exposed to a light–dark cycles for 12 h to promote conidia release. The conidia were harvested by washing the media surfaces with ddH_2_O, filtering through lens-cleaning paper, and adjusting to 2 mL to ensure uniform distribution.

To observe the conidiophores, hyphae were scraped onto the media surfaces, and the media blocks were transferred to microscope slides. These slides were incubated at 28 °C under light conditions to allow conidiophore development. Conidiophore formation and morphology were examined under a light microscope at 24 h post-incubation (hpi).

### Appressorium formation and turgo pressure assays

For appressorium formation assays, 20 μL of spore suspensions (5 × 10^4^ spores per mL) were spotted onto hydrophobic coverslips and incubated at 28℃ under humified, dark conditions for 4, 8, and 24 h. Over 100 conidia were examined per strain, with consistent results obtained from at least three biological replicates, each containing three technical replicates.

Appressorium turgor pressure was assessed by treating the appressoriia with 1, 2, 3, and 4 M glycerin solution for 5 min, followed by observation under a light microscope. At least 100 conidia/appressoria were observed per replicate, and the experiments were repeated three time with three technical replicates.

For lipid staining, the conidia were stained with 10 µg/mL Nile red.

### Plant infection and penetration assays

For hyphae-mediated infection assays, the strains were first cultured in liquid CM at 28℃ under constant shaking at 120 rpm for 3 days. Mycelia were harvested by filtration, rinsed with sterile ddH_2_O, and used to inoculate intact and injured barley leaves. The inoculated barely were kept in a dark and humified environment for 24 h, then transferred to a growth chamber under a 12–12 h light–dark cycle for 5 days.

For conidia-mediated infection assays, spore suspensions (5 × 10^4^ spores per mL containing 0.02% Tween-20) were used to inoculate 7-day-old intact and injured barley leaves and sprayed on 3-week-old rice seedlings (cv. CO39). The seedlings were kept in a dark and humified environment for 24 h, then moved to a growth chamber under a 12–12 h light–dark cycle for 5 days.

For penetration assays, spore suspensions without Tween-20 were infiltrated into the leaf sheaths of 4-week-old rice, and kept in a dark and humid condition for 24 h. Development of invasive hyphae was observed under a light microscope.

### Co-localization assay

To investigate the subcellular localization of MoNeo1 in *M. oryzae*, MoKex2-mCherry, MoSnc1 and MoVps35-mScarlet were utilized as previously reported. Vector pairs expressing MoNeo1-GFP/MoKex2-mCherry, MoNeo1-GFP/MoVps35-mScarlet and GFP-MoSnc1/MoNeo1-mCherry, respectively, were co-transformed into the Δ*Moneo1* mutant protoplasts, respectively. Transformants were screened by PCR and confirmed by confocal microscopy using GFP signal detection. Co-localization study The strains co-expressing the tagged proteins were generated and analyzed as previously described.

### Affinity purification and mass spectrometric analysis

For affinity purification, mycelia of RP27-Flag and MoNeo1-Flag strains were harvested, cryogenically powdered in liquid nitrogen, and lysed in extraction buffer (10 mM Tris–HCl, pH 7.5; 150 mM NaCl; 0.5 mM EDTA; 1% Triton X-100; 2 mM PMSF) supplemented with protease inhibitor cocktail (Cat. no. C510026; Sangon Biotech, Shanghai, China) for 30 min. Following lysis, total proteins were incubated with 30 μL of Anti-Flag Magarose beads (Cat. no. SM00901; Smart-Lifesciences, China) at 4 °C for 4 h. Bound proteins were subsequently eluted by heating at 100 °C for 10 min. Mass spectrometric analysis was performed as previously described (Zheng et al. 2018).

### Co-immunoprecipitation assay

For Co-IP assays, the strains expressing the fusion proteins were cultured in liquid CM at 28℃, 110 rpm for 3 d. The samples were collected and ground into fine powder in liquid nitrogen and lysed in lysis buffer for 30 min. Total cell lysates were incubated with GFP-Trap beads at 4 ℃ for 4 h. A magnetic frame was used to further wash the beads three times with 500 μL cold washing buffer (50 mM Tris, 0.15 M NaCl, and pH 7.4). Bound proteins were eluted by heating at 100℃ for 15 min with protein loading buffer and analyzed by western blot using anti-GFP antibodies (Cat. M20004M, Abmart, Shanghai, China).

### Lipid extraction and analysis

Mycelia were cultured in liquid CM at 28 ºC under constant shaking at 120 rpm for 4 days. The samples were heated at 75 ºC for 15 min with isopropanol containing 0.05% (v/v) butylated hydroxytoluene (BHT) (Sigma-Aldrich, Saint Louis, MI, USA). Total lipid extraction was performed using chloroform/methanol (2:1) containing 0.01% BHT. After adding 1 M KCl and H_2_O, the mixture was centrifuged and the upper phase was discarded. The solvent was evaporated under N_2_ gas, and the residue was redissolved in chloroform (5 mg/mL).

Lipidome analysis was conducted using an Agilent HPLC system coupled with an Applied Biosystems Triple Quadrupole/Ion Trap 4000 QTrap mass spectrometer (Applied Biosystems, USA) (Zhao et al., 2022).

### Microscopy examinations

A Nikon Ci-S fluorescence microscope and a Nikon CUS-W1 spinning-disk confocal microscope (Japan) were used to observe GFP and mCherry fluorescence, with emission and excitation wavelengths of 488 nm and 561 nm.

## Supplementary Information

Supplementary Material 1.

Supplementary Material 2.

Supplementary Material 3.

## Data Availability

All data and materials are available in the paper and online supplemental files.

## Abbreviations

CO: Conidia
CP: Conidiophore
PI4P: Phosphatidylinositol 4-phosphate
PS: Phosphatidylserine
PE: Phosphatidylethanolamine
TGN: Trans-Gologi network
PA: Phosphatidic acid
PC: Phosphatidylcholine
PI: Phosphatidylinositol
LPE: Lysophosphatidylethanolamine
LPG: Lysophosphatidylglycerol
TAG: Triacylglycerol
DAG: Diacylglycerol
CM: Complete media
RBM: Rice bran media
CFW: Calcofluor White
CR: Conge Red
HPLC-MS: High-performance liquid chromatography-mass spectrometry
ID: Intellectual disability
ADHD: Attention deficit hyperactivity disorder
HPI: Hours post-incubation
BHT: Butylated hydroxytoluene

## References

[CR1] Andersen JP, Vestergaard AL, Mikkelsen SA, Mogensen LS, Chalat M, Molday RS (2016) P4-ATPases as phospholipid flippases—structure, function, and enigmas. Front Physiol 7:275. 10.3389/fphys.2016.00275 27458383 10.3389/fphys.2016.00275 PMC4937031

[CR2] Bai L, Jain BK, You Q, Duan HD, Takar M, Graham TR, Li H (2021) Structural basis of the P4B ATPase lipid flippase activity. Nat Commun 12:5963. 10.1038/s41467-021-26273-0 34645814 10.1038/s41467-021-26273-0 PMC8514546

[CR3] Baldridge RD, Graham TR (2012) Identification of residues defining phospholipid flippase substrate specificity of type IV P-type ATPases. Proc Natl Acad Sci U S A 109:290–298. 10.1073/pnas.1115725109 10.1073/pnas.1115725109 PMC3277569 22308393

[CR4] Balhadère PV, Talbot NJ (2001) PDE1 encodes a P-type ATPase involved in appressorium-mediated plant infection by the rice blast fungus *Magnaporthe grisea*. Plant Cell 13:1987–2004. 10.1105/tpc.010056 11549759 10.1105/TPC.010056 PMC139447

[CR5] Bard F, Malhotra V (2006) The formation of TGN-to-plasma-membrane transport carriers. Annu Rev Cell Dev Biol 22:439–455. 10.1146/annurev.cellbio.21.012704.133126 16824007 10.1146/annurev.cellbio.21.012704.133126

[CR6] Bonifacino JS, Glick BS (2004) The mechanisms of vesicle budding and fusion. Cell 116:153–166. 10.1016/S0092-8674(03)01079-1 14744428 10.1016/s0092-8674(03)01079-1

[CR7] Chen X, Selvaraj P, Lin L, Fang W, Wu C, Yang P, Zhang J, Abubakar YS, Yang F, Lu G, Liu W, Wang Z, Naqvi NI, Zheng W (2023) Rab7/Retromer‐based endolysosomal trafficking is essential for proper host invasion in rice blast. New Phytol 239:1384–1403. 10.1111/nph.19050 37291895 10.1111/nph.19050

[CR8] Chen X, Hu J, Zhong H, Wu Q, Fang Z, Cai Y, Huang P, Abubakar YS, Zhou J, Naqvi NI, Wang Z, Zheng W (2024) Vacuolar recruitment of retromer by a SNARE complex enables infection‐related trafficking in rice blast. New Phytol 244:997–1012. 10.1111/nph.20069 39180241 10.1111/nph.20069

[CR9] Cullen PJ, Steinberg F (2018) To degrade or not to degrade: mechanisms and significance of endocytic recycling. Nat Rev Mol Cell Biol 19:679–696. 10.1038/s41580-018-0053-7 30194414 10.1038/s41580-018-0053-7

[CR10] Daleke DL (2007) Phospholipid flippases. J Biol Chem 282:821–825. 10.1074/jbc.R600035200 17130120 10.1074/jbc.R600035200

[CR11] Dalton LE, Bean BDM, Davey M, Conibear E (2017) Quantitative high-content imaging identifies novel regulators of Neo1 trafficking at endosomes. Mol Biol Cell 28:1539–1550. 10.1091/mbc.e16-11-0772 28404745 10.1091/mbc.E16-11-0772 PMC5449152

[CR12] Gu F, Crump CM, Thomas G (2001) Trans-Golgi network sorting. Cell Mol Life Sci 58:1067–1084. 10.1007/PL00000922 11529500 10.1007/PL00000922 PMC1424219

[CR13] Hankins HM, Baldridge RD, Xu P, Graham TR (2015) Role of flippases, scramblases and transfer proteins in phosphatidylserine subcellular distribution. Traffic 16:35–47. 10.1111/tra.12233 25284293 10.1111/tra.12233 PMC4275391

[CR14] Hiraizumi M, Yamashita K, Nishizawa T, Nureki O (2019) Cryo-EM structures capture the transport cycle of the P4-ATPase flippase. Science 365:1149–1155. 10.1126/science.aay3353 31416931 10.1126/science.aay3353

[CR15] Hu G, Kronstad JW (2010) A putative P-type ATPase, Apt1, is involved in stress tolerance and virulence in *Cryptococcus neoformans*. Eukaryot Cell 9:74–83. 10.1128/EC.00289-09 19949048 10.1128/EC.00289-09 PMC2805298

[CR16] Hua Z, Fatheddin P, Graham TR (2002) An essential subfamily of Drs2p-related P-type ATPases is required for protein trafficking between Golgi complex and endosomal/vacuolar system. Mol Biol Cell 13:3162–3177. 10.1091/mbc.e02-03-0172 12221123 10.1091/mbc.E02-03-0172 PMC124150

[CR17] Jain BK, Duan HD, Valentine C, Samiha A, Li H, Graham TR (2025) P4-ATPases control phosphoinositide membrane asymmetry and neomycin resistance. Nat Cell Biol 27:1114–1124. 10.1038/s41556-025-01692-z 40646185 10.1038/s41556-025-01692-z PMC12270916

[CR18] Kohlwein SD (2010) Triacylglycerol homeostasis: insights from yeast. J Biol Chem 285:15663–15667. 10.1074/jbc.R110.118356 20231294 10.1074/jbc.R110.118356 PMC2871431

[CR19] Kvainickas A, Jimenez-Orgaz A, Nägele H, Hu Z, Dengjel J, Steinberg F (2017) Cargo-selective SNX-BAR proteins mediate retromer trimer independent retrograde transport. J Cell Biol 216:3677–3693. 10.1083/jcb.201702137 28935632 10.1083/jcb.201702137 PMC5674888

[CR20] Maxfield FR, McGraw TE (2004) Endocytic recycling. Nat Rev Mol Cell Biol 5:121–132. 10.1038/nrm1315 15040445 10.1038/nrm1315

[CR21] Meng T, Chen X, He Z, Huang H, Lin S, Liu K, Bai G, Liu H, Xu M, Zhuang H, Zhang Y, Waqas A, Liu Q, Zhang C, Sun XD, Huang H, Umair M, Yan Y, Feng D (2023) ATP9A deficiency causes ADHD and aberrant endosomal recycling via modulating RAB5 and RAB11 activity. Mol Psychiatry 28:1219–1231. 10.1038/s41380-022-01940-w 36604604 10.1038/s41380-022-01940-w PMC9816018

[CR22] Murray DH, Tamm LK (2011) Molecular mechanism of cholesterol- and polyphosphoinositide-mediated syntaxin clustering. Biochemistry 50:9014–9022. 10.1021/bi201307u 21916482 10.1021/bi201307u PMC3199950

[CR23] Muthusamy B-P, Natarajan P, Zhou X, Graham TR (2009) Linking phospholipid flippases to vesicle-mediated protein transport. Biochim Biophys Acta BBA - Mol Cell Biol Lipids 1791:612–619. 10.1016/j.bbalip.2009.03.004 10.1016/j.bbalip.2009.03.004 PMC3770137 19286470

[CR24] Nalley L, Tsiboe F, Durand-Morat A, Shew A, Thoma G (2016) economic and environmental impact of rice blast pathogen (*Magnaporthe oryzae*) alleviation in the United States. PLoS ONE 11:e0167295. 10.1371/journal.pone.0167295 27907101 10.1371/journal.pone.0167295 PMC5131998

[CR25] Norris AC, Mansueto AJ, Jimenez M, Yazlovitskaya EM, Jain BK, Graham TR (2024) Flipping the script: advances in understanding how and why P4-ATPases flip lipid across membranes. Biochim Biophys Acta (BBA) - Mol Cell Res 1871:119700. 10.1016/j.bbamcr.2024.119700 10.1016/j.bbamcr.2024.119700 PMC12424579 38382846

[CR26] Oliveira‐Garcia E, Yan X, Oses‐Ruiz M, de Paula S, Talbot NJ (2024) Effector‐triggered susceptibility by the rice blast fungus *Magnaporthe oryzae*. New Phytol 241:1007–1020. 10.1111/nph.19446 38073141 10.1111/nph.19446

[CR27] Pomorski T, Menon AK (2006) Lipid flippases and their biological functions. Cell Mol Life Sci 63:2908–2921. 10.1007/s00018-006-6167-7 17103115 10.1007/s00018-006-6167-7 PMC11136118

[CR28] Poulsen LR, López-Marqués RL, McDowell SC, Okkeri J, Licht D, Schulz A, Pomorski T, Harper JF, Palmgren MG (2008) The Arabidopsis P4-ATPase ALA3 localizes to the Golgi and requires a β-Subunit to function in lipid translocation and secretory vesicle formation. Plant Cell 20:658–676. 10.1105/tpc.107.054767 18344284 10.1105/tpc.107.054767 PMC2329932

[CR29] Prezant TR, Chaltraw WE Jr, Fischel-Ghodsian N (1996) Identification of an overexpressed yeast gene which prevents aminoglycoside toxicity. Microbiology (Reading) 142:3407–3414. 10.1099/13500872-142-12-3407 10.1099/13500872-142-12-3407 9004503

[CR30] Rizzo J, Oliveira DL, Joffe LS, Hu G, Gazos-Lopes F, Fonseca FL, Almeida IC, Frases S, Kronstad JW, Rodrigues ML (2014) Role of the Apt1 protein in polysaccharide secretion by *Cryptococcus neoformans*. Eukaryot Cell 13:715–726. 10.1128/EC.00273-13 24337112 10.1128/EC.00273-13 PMC4054279

[CR31] Savary S, Willocquet L, Pethybridge SJ, Esker P, McRoberts N, Nelson A (2019) The global burden of pathogens and pests on major food crops. Nat Ecol Evol 3:430–439. 10.1038/s41559-018-0793-y 30718852 10.1038/s41559-018-0793-y

[CR32] Schultzhaus Z, Cunningham GA, Mouriño-Pérez RR, Shaw BD (2019) The phospholipid flippase DnfD localizes to late Golgi and is involved in asexual differentiation in *Aspergillus nidulans*. Mycologia 111:13–25. 10.1080/00275514.2018.1543927 30699058 10.1080/00275514.2018.1543927

[CR33] Scott CC, Vacca F, Gruenberg J (2014) Endosome maturation, transport and functions. Semin Cell Dev Biol 31:2–10. 10.1016/j.semcdb.2014.03.034 24709024 10.1016/j.semcdb.2014.03.034

[CR34] Sebastian TT, Baldridge RD, Xu P, Graham TR (2012) Phospholipid flippases: building asymmetric membranes and transport vesicles. Biochim Biophys Acta BBA Mol Cell Biol Lipids 1821:1068–1077. 10.1016/j.bbalip.2011.12.007 10.1016/j.bbalip.2011.12.007 PMC3368091 22234261

[CR35] Simonetti B, Paul B, Chaudhari K, Weeratunga S, Steinberg F, Gorla M, Heesom KJ, Bashaw GJ, Collins BM, Cullen PJ (2019) Molecular identification of a BAR domain-containing coat complex for endosomal recycling of transmembrane proteins. Nat Cell Biol 21:1219–1233. 10.1038/s41556-019-0393-3 31576058 10.1038/s41556-019-0393-3 PMC6778059

[CR36] Sorkin A, von Zastrow M (2009) Endocytosis and signalling: intertwining molecular networks. Nat Rev Mol Cell Biol 10:609–622. 10.1038/nrm2748 19696798 10.1038/nrm2748 PMC2895425

[CR37] Takamori S, Holt M, Stenius K, Lemke EA, Grønborg M, Riedel D, Urlaub H, Schenck S, Brügger B, Ringler P, Müller SA, Rammner B, Gräter F, Hub JS, De Groot BL, Mieskes G, Moriyama Y, Klingauf J, Grubmüller H, Heuser J, Wieland F, Jahn R (2006) Molecular anatomy of a trafficking organelle. Cell 127:831–846. 10.1016/j.cell.2006.10.030 17110340 10.1016/j.cell.2006.10.030

[CR38] Takar M, Wu Y, Graham TR (2016) The essential Neo1 protein from budding yeast plays a role in establishing aminophospholipid asymmetry of the plasma membrane. J Biol Chem 291:15727–15739. 10.1074/jbc.M115.686253 27235400 10.1074/jbc.M115.686253 PMC4957055

[CR39] Takatsu H, Baba K, Shima T, Umino H, Kato U, Umeda M, Nakayama K, Shin HW (2011) ATP9B, a P4-ATPase (a putative aminophospholipid translocase), localizes to the trans-Golgi network in a CDC50 protein-independent manner. J Biol Chem 286:38159–38167. 10.1074/jbc.M111.281006 21914794 10.1074/jbc.M111.281006 PMC3207472

[CR40] Tanaka Y, Ono N, Shima T, Tanaka G, Katoh Y, Nakayama K, Takatsu H, Shin HW (2016) The phospholipid flippase ATP9A is required for the recycling pathway from the endosomes to the plasma membrane. Mol Biol Cell 27:3883–3893. 10.1091/mbc.E16-08-0586 27733620 10.1091/mbc.E16-08-0586 PMC5170610

[CR41] Timcenko M, Lyons JA, Januliene D, Ulstrup JJ, Dieudonné T, Montigny C, Ash MR, Karlsen JL, Boesen T, Kühlbrandt W, Lenoir G, Moeller A, Nissen P (2019) Structure and autoregulation of a P4-ATPase lipid flippase. Nature 571:366–370. 10.1038/s41586-019-1344-7 31243363 10.1038/s41586-019-1344-7

[CR42] Tu Y, Seaman MNJ (2021) Navigating the controversies of retromer-mediated endosomal protein sorting. Front Cell Dev Biol 9:658741. 10.3389/fcell.2021.658741 34222232 10.3389/fcell.2021.658741 PMC8247582

[CR43] Van der Mark V, Elferink R, Paulusma C (2013) P4 ATPases: flippases in health and disease. Int J Mol Sci 14:7897–7922. 10.3390/ijms14047897 23579954 10.3390/ijms14047897 PMC3645723

[CR44] Wehman AM, Poggioli C, Schweinsberg P, Grant BD, Nance J (2011) The P4-ATPase TAT-5 inhibits the budding of extracellular vesicles in *C.* *elegans embryos*. Curr Biol 21:1951–1959. 10.1016/j.cub.2011.10.040 22100064 10.1016/j.cub.2011.10.040 PMC3237752

[CR45] Wu Y, Takar M, Cuentas-Condori AA, Graham TR (2016) Neo1 and phosphatidylethanolamine contribute to vacuole membrane fusion in *Saccharomyces cerevisiae*. Cell Logist 6:e1228791. 10.1080/21592799.2016.1228791 27738552 10.1080/21592799.2016.1228791 PMC5058351

[CR46] Yagi T, Nakabuchi R, Muranaka Y, Tanaka G, Katoh Y, Nakayama K, Takatsu H, Shin HW (2025) Lipid flippases ATP9A and ATP9B form a complex and contribute to the exocytic pathway from the Golgi. Life Sci Alliance 8:e202403163. 10.26508/lsa.202403163 40234049 10.26508/lsa.202403163 PMC12000689

[CR47] Yun Y, Guo P, Zhang J, You H, Guo P, Deng H, Hao Y, Zhang L, Wang X, Abubakar YS, Zhou J, Lu G, Wang Z, Zheng W (2020) Flippases play specific but distinct roles in the development, pathogenicity, and secondary metabolism of *Fusarium graminearum*. Mol Plant Pathol 21:1307–1321. 10.1111/mpp.12985 32881238 10.1111/mpp.12985 PMC7488471

[CR48] Zhang D, Hu J, Hong Y, Fan Y, Peng M, Abubakar YS, Liang S, Jiang N, Lin L, Pan X, Zheng H, Naqvi NI, Wang Z, Zheng W (2025) Retromer regulates macro‐ and micro‐autophagy via distinct vacuolar proteases in the rice blast fungus. Adv Sci e10068. 10.1002/advs.202510068 10.1002/advs.202510068 PMC12591164 40842061

[CR49] Zhang H, Yang J, Liu M, Xu X, Yang L, Liu X, Peng Y, Zhang Z (2024) Early molecular events in the interaction between *Magnaporthe oryzae* and rice. Phytopathol Res 6:9. 10.1186/s42483-024-00226-z

[CR50] Zhao J, Sun P, Sun Q et al (2022) The MoPah1 phosphatidate phosphatase is involved in lipid metabolism, development, and pathogenesis in Magnaporthe oryzae. Mol Plant Pathol 23:720–732. 10.1111/mpp.13193 10.1111/mpp.13193 PMC8995063 35191164

[CR51] Zheng W, Lin Y, Fang W, Zhao X, Lou Y, Wang G, Zheng H, Liang Q, Abubakar YS, Olsson S, Zhou J, Wang Z (2018) The endosomal recycling of FgSnc1 by FgSnx41–FgSnx4 heterodimer is essential for polarized growth and pathogenicity in *Fusarium graminearum*. New Phytol 219:654–671. 10.1111/nph.15178 29676464 10.1111/nph.15178

