# Supplementary material for: The lipid flippase MoNeo1 mediates vesicle trafficking and pathogenicity in Magnaporthe oryzae
Source: Stress Biol. 2026 Apr 13;6(1):28. doi: 10.1007/s44154-026-00305-5 (PMC13076854; doi:10.1007/s44154-026-00305-5)
Supplement: Supplementary file 1 — Supplementary Material 1. [file 44154_2026_305_MOESM1_ESM.docx]

**The lipid flippase MoNeo1 mediates vesicle trafficking and pathogenicity in *Magnaporthe oryzae***

Yan Cai^1,2^, Xiuwei Huang^1,2^, Yufan Nie^1,2^, Yueying Luan^1,2^, Aarti Aarti^1,2^, Qing Gong^1,2^, Peng Sun^3^, Yakubu Saddeeq Abubakar^1,4^, Baohua Wang^1,2^, Airong Wang^1,2^, Guotian Li^5^, Lili Lin^1,2^*, Wenhui Zheng^1,2^*

^1^State Key Laboratory for Ecological Pest Control of Fujian and Taiwan Crops, College of Plant Protection, Fujian Agriculture and Forestry University, Fuzhou 350002, China;

^2^Key Laboratory of Bio-pesticide and Chemistry Biology, Ministry of Education, College of Plant Protection, Fujian Agriculture and Forestry University, Fuzhou 350002, China;

^3^State Key Laboratory for Biology of Plant Diseases and Insect Pests—Key Laboratory of Control of Biological Hazard Factors (Plant Origin) for Agri-Product Quality and Safety, Ministry of Agriculture, Institute of Plant Protection, Chinese Academy of Agricultural Sciences, Beijing 100081, China;

^4^Department of Biochemistry, Faculty of Life Sciences, Ahmadu Bello University, Zaria 810281, Nigeria;

^5^National Key Laboratory of Agricultural Microbiology, Hubei Hongshan Laboratory, Hubei Key Laboratory of Plant Pathology, The Center of Crop Nanotechnology, Huazhong Agricultural University, Wuhan 430070, China.

* Correspondence: Wenhui Zheng (wenhuiz@126.com); Lili Lin (lilly116@163.com)

**Supplemental information**


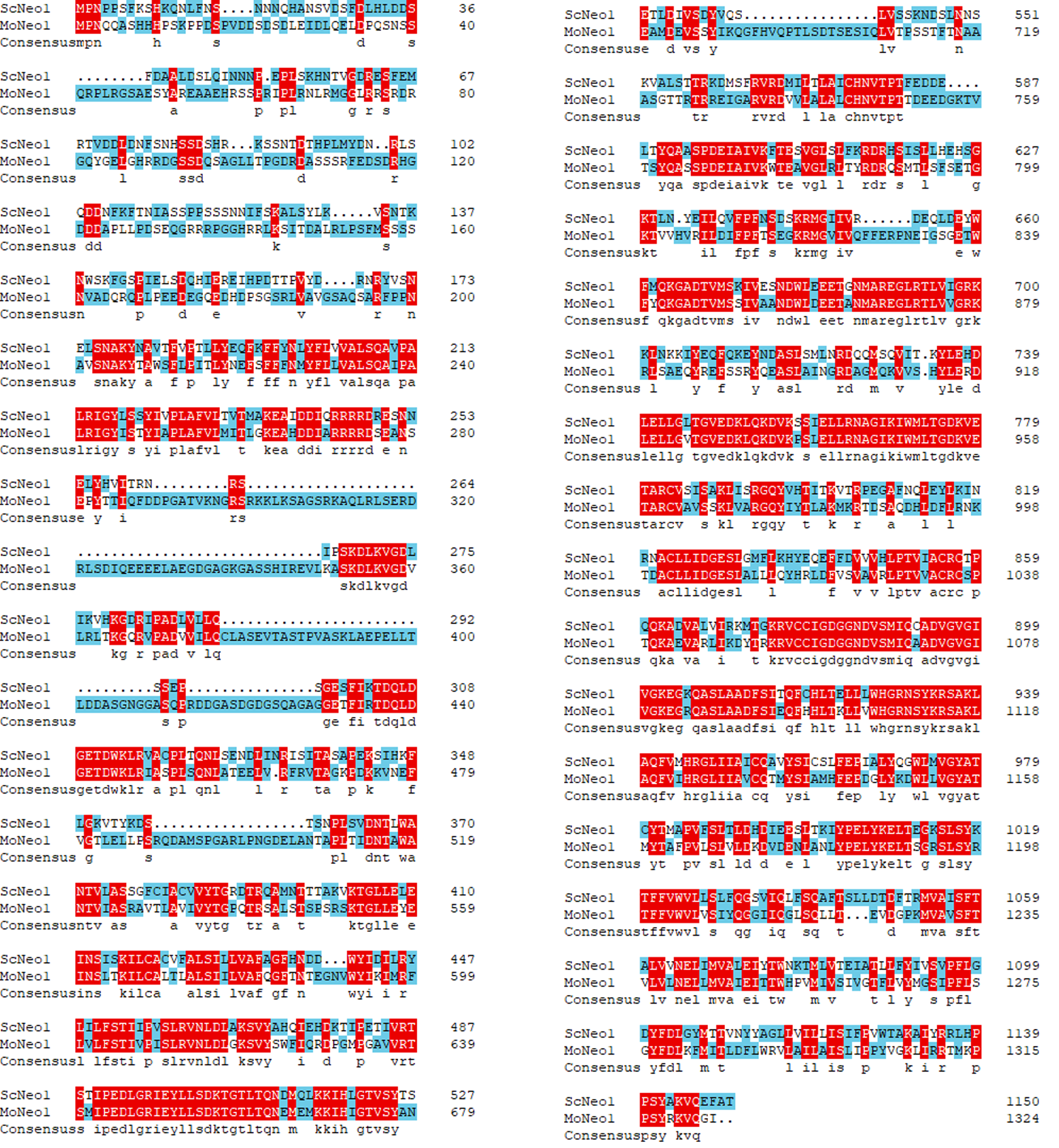


**Fig. S1** Alignment of amino acid sequences between ScNeo1 and MoNeo1.


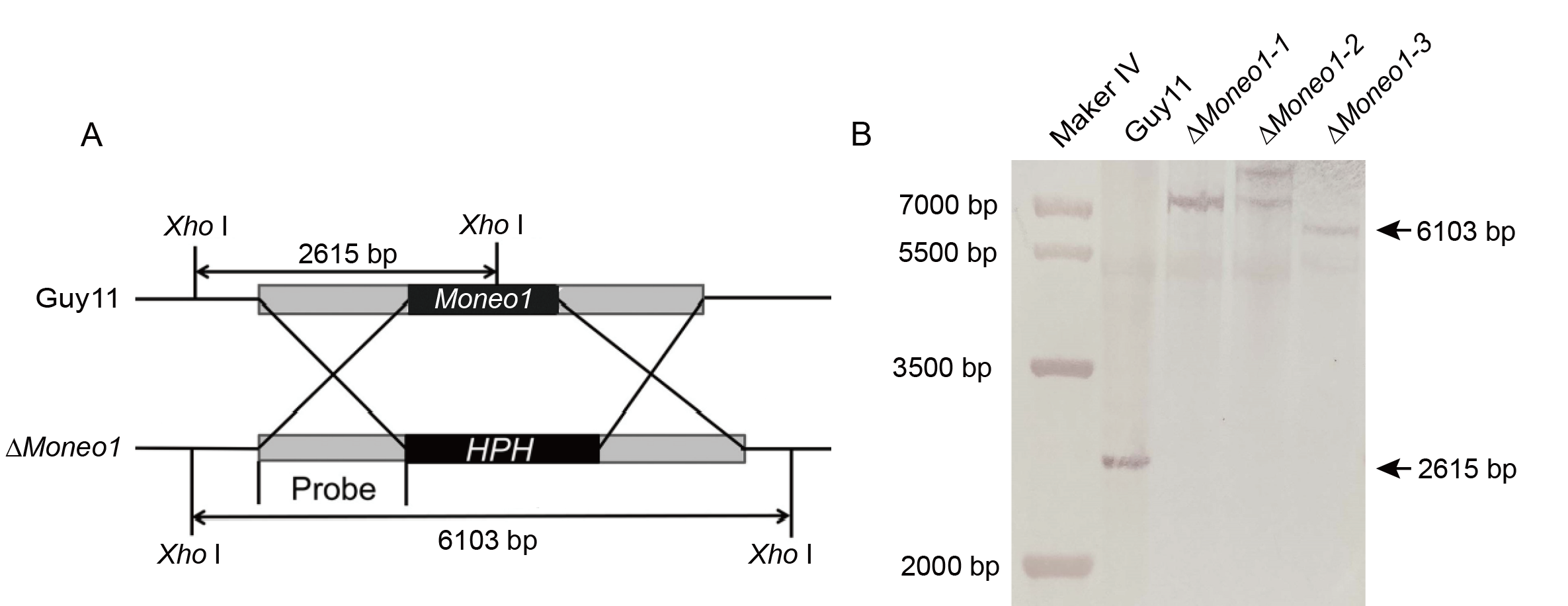


**Fig. S2** Targeted gene deletion of *MoNEO1* in *M. oryzae.* **A** Schematic diagram of the targeted gene disruption strategy used to generate Δ*Moneo1* strains via homologous recombination. **B** Southern blot analysis confirming the successful replacement of *MoNEO1* with hygromycin resistance gene (hph) in the Δ*Moneo1* strains via single-crossover recombination.


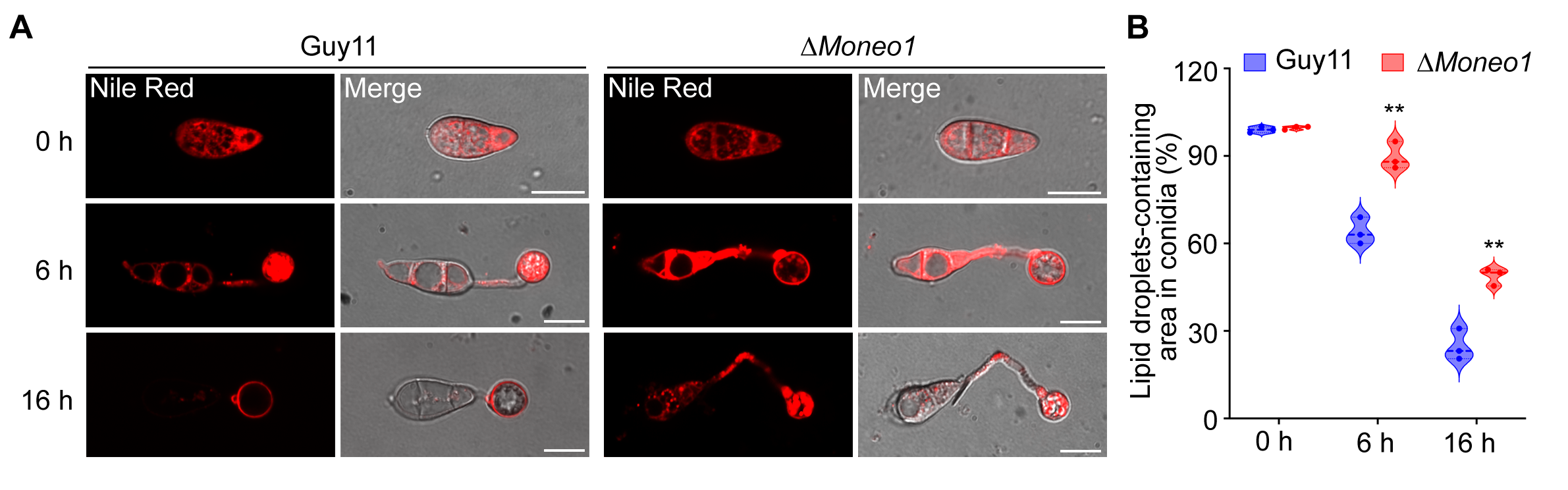


Fig. S3 Lipid droplet staining during appressorium development. **A** Nile red staining of lipid droplets in conidia and appressoria at 0, 6, and 16 h post-induction in Guy11 and Δ*Moneo1*. **B** Quantification of lipid droplet-containing area in conidia. Scale bars, 10 μm. **, P < 0.01.


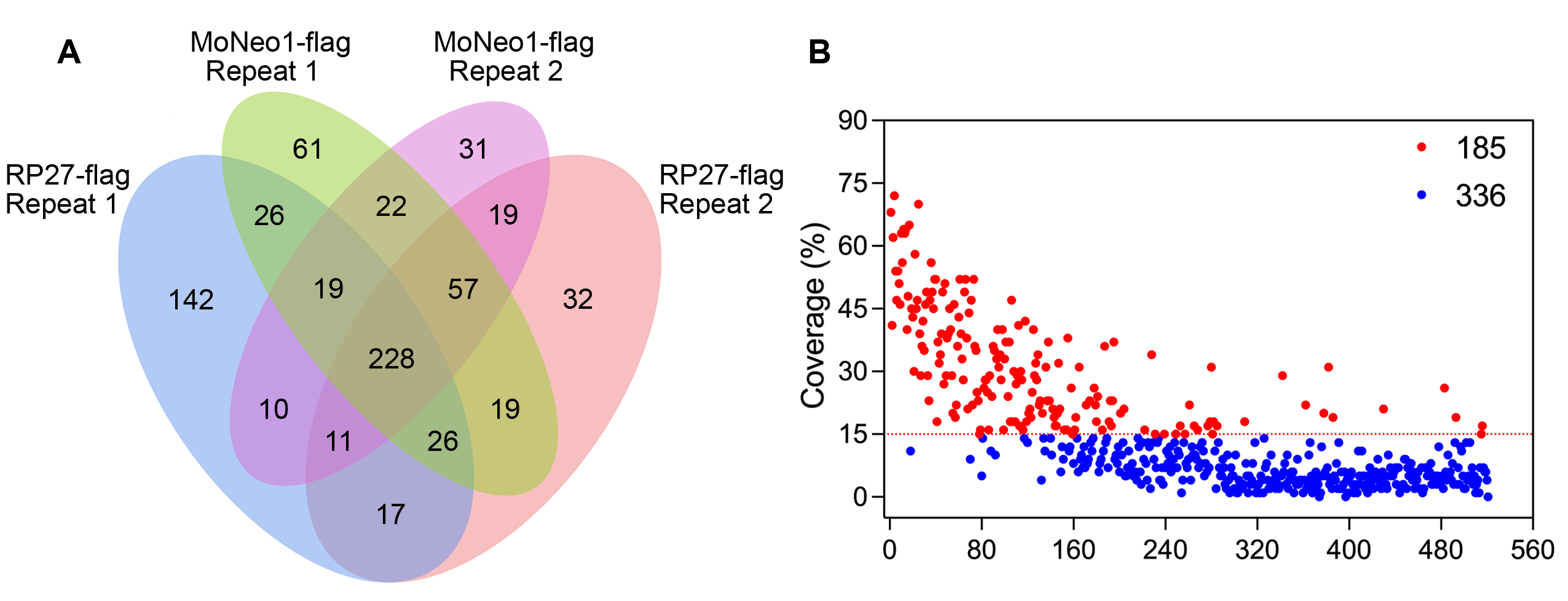


**Fig. S4** Identification and coverage analysis of MoNeo1-associated proteins. **A** Venn diagram illustrating the overlap of proteins identified in MoNeo1-flag and RP27-flag immunoprecipitation experiments across two biological replicates. **B** Scatter plot showing the sequence coverage percentage of proteins identified in the MoNeo1-flag pull-down assay. Proteins with coverage ≥15% (red) were selected for further analysis, whereas those with lower coverage (blue) were excluded. The dashed line marks the 15% coverage threshold.

**Video S1** Dynamics of MoNeo1-GFP in the vegetative hyphae of *M. oryzae*.

**Table S1. The primers used in this study**

| Primers | Sequences(5’ → 3’) | Application |
| --- | --- | --- |
| *MoNEO1*-AF | GAACAAAAGCTGGGTCGCTGGAGCATTGTTGAT | *MoNEO1* deletion |
| *MoNEO1*-AR | CAGCGGCGCGCCGAACGACTGGCTTTAGGCTTTACT |  |
| *MoNEO1*-BF | ACCGGGCCGGCCGGACATTGGCGGGTGTTTAGT |  |
| *MoNEO1*-BR | GGTGGCGGCCGCTCTGCAGAAGGGAGAAGGGTG |  |
| YG/F | GATGTAGGAGGGCGTGGATATGTCCT |  |
| HY/R | GTATTGACCGATTCCTTGCGGTCCGAA |  |
| HYG/F | GGCTTGGCTGGAGCTAGTGGAGGTCAA |  |
| HYG/R | AACCCGCGGTCGGCATCTACTCTATTC |  |
| *MoNEO1*-OF | AAAGGTCGTGTCGCATTA | Δ*Moneo1* mutant screen |
| *MoNEO1*-OR | TGGCACTCCGCTTGTAG |  |
| *MoNEO1*-UA | CAACCAACTGCGGAGATA |  |
| H853-R | GACAGACGTCGCGGTGAGTT |  |
| MoNeo1-GF | AGGGAACAAAAGCTGGGTACCGCTTTCGGCGGCATACT | MoNeo1-GFP  generation |
| MoNeo1-GR | GCCCTTGCTCACCATAAGCTTTATACCTTGCACCTTTCTGT |  |
| MoNeo1-pGF | GGGTACCGGGCCCCCCCTCGAGCGGTTTCCTGGAGTCAC | GFP-MoNeo1 generation |
| MoNeo1-pGR | AGTTCCTCGCCCTTGCCCATCCCGCATTTATCGATGTCTA |  |
| Neo1-GO-F | GCATGGATGAACTCTACAAGATGCCGAACCAACAGG |  |
| Neo1-GO-R | CCCCCGGGCTGCAGGAATTCTTATATACCTTGCACCTTTC |  |
| GFP-F | ATGGGCAAGGGCGAGGAACTGT |  |
| GFP-R | CTTGTAGAGTTCATCCATGCCAT |  |
| Neo1- mCF | TATAGGGCGAATTGGGTACCGCTTTCGGCGGCATACT | MoNeo1-mCherry generation |
| Neo1- mCR | CCCTTGCTCACCATAAGCTTTATACCTTGCACCTTTCTGT |  |
| pKNT-Neo1-flag | GGGTACCGGGCCCCCCCTCGAGGCTTTCGGCG  GCATACT | Co-IP |
| pKNT-Neo1-flag | TGTAGTCCATACCACCATCGATTATACCTTGCACC  TTTCTGT |  |
